# Hepatitis C Virus Protects Human B Lymphocytes from Fas-Mediated Apoptosis via E2-CD81 Engagement

**DOI:** 10.1371/journal.pone.0018933

**Published:** 2011-04-19

**Authors:** Zhihui Chen, Yongzhe Zhu, Yanli Ren, Yimin Tong, Xian Hua, Fenghui Zhu, Libin Huang, Yuan Liu, Yuan Luo, Wei Lu, Ping Zhao, Zhongtian Qi

**Affiliations:** 1 Department of Microbiology, Shanghai Key Laboratory of Medical Biodefense, Second Military Medical University, Shanghai, China; 2 Department of Infectious Diseases, Affiliated Changhai Hospital, Second Military Medical University, Shanghai, China; 3 Department of Epidemiology, Second Military Medical University, Shanghai, China; University of Kansas Medical Center, United States of America

## Abstract

HCV infection is often associated with B-cell regulatory control disturbance and delayed appearance of neutralizing antibodies. CD81 is a cellular receptor for HCV and can bind to HCV envelope protein 2 (E2). CD81 also participates to form a B cell costimulatory complex. To investigate whether HCV influences B cell activation and immune function through E2 -CD81 engagement, here, human Burkitt's lymphoma cell line Raji cells and primary human B lymphocytes (PHB) were treated with HCV E2 protein and cell culture produced HCV particles (HCVcc), and then the related cell phenotypes were assayed. The results showed that both E2 and HCVcc triggered phosphorylation of IκBα, enhanced the expression of anti-apoptosis Bcl-2 family proteins, and protected Raji cells and PHB cells from Fas-mediated death. In addition, both E2 protein and HCVcc increased the expression of costimulatory molecules CD80, CD86 and CD81 itself, and decreased the expression of complement receptor CD21. The effects were dependent on E2-CD81 interaction on the cell surface, since CD81-silenced Raji cells did not respond to both treatments; and an E2 mutant that lose the CD81 binding activity, could not trigger the responses of both Raji cells and PHB cells. The effects were not associated with HCV replication in cells, for HCV pseudoparticle (HCVpp) and HCVcc failed to infect Raji cells. Hence, E2-CD81 engagement may contribute to HCV-associated B cell lymphoproliferative disorders and insufficient neutralizing antibody production.

## Introduction

Hepatitis C virus (HCV)infection is an important cause of chronic liver diseases, including chronic hepatitis, liver cirrhosis and hepatocellular carcinoma [1]. HCV is an enveloped virus classified in the Flaviviridae family. The HCV envelope proteins consist of two heavily glycosylated proteins, E1 and E2, which act as the ligands for cellular receptors [2]. Human CD81 is the first identified necessary receptor for HCV cell entry, which can directly bind with HCV E2 protein [3], [4]. CD81 is a widely distributed cell-surface tetraspanin that participates in different molecular complexes on various cell types, including hepatocytes, B lymphocytes, T lymphocytes and natural killer cells [5]. It has been proposed that HCV exploits CD81 not only to invade hepatocytes but also to modulate the host immune responses. It was reported that cross-linking of CD81 by HCV E2 protein could activate human T cells and inhibit human NK cells *in vitro* [6], [7]. On B cell, CD81 is known to form B cell costimulatory complex with CD19, CD21, and interferon-inducible Leu-13 (CD225) proteins [8]. This complex reduces the threshold for B cell activation via the B cell receptor by bridging antigen specific recognition and CD21-mediated complement recognition [9].

HCV infection is often associated with B-cell lymphoproliferative disorders such as mixed cryoglobulinemia (MC) and non-Hodgkin lymphoma (NHL) [10], [11]. Reports showing the clinical resolution of MC and lymphomas after successful interferon antiviral treatment suggest an important pathogenic role for HCV in B-cell dysfunction [12], [13]. It was reported that engagement of CD81 on human B cells by a combination of HCV E2 protein and anti-CD81 mAb leads to the proliferation of naïve B cells, and E2-CD81 interaction induces protein tyrosine phosphorylation and hypermutation of the immunoglobulin genes in B cell lines [14], [15], [16]. These data suggest that E2 protein should play a role in the development of B-cell pathophysiology, but the underlying mechanisms remain unclear.

E2 protein is the main target of HCV neutralizing antibodies [17], [18]. The neutralizing antibodies can block HCV infection via interruption of viral attachment, entry or membrane fusion, and have been considered to play an important role in prevention and possibly recovery from HCV infection [19], [20]. However, neutralizing antibodies are typically delayed in appearance in acute HCV infection, generally do not confer protective immunity [18], [21]. The chimpanzee is the only available animal model that could be naturally infected by HCV, the majority of infected chimpanzees developed a low titer neutralizing antibodies response late in disease, which failed to associate with viral clearance [21], [22].The reasons for this need to be addressed.

In the present study, with the use of HCV E2 protein and cell culture produced HCV (HCVcc) to engage CD81 on surface of Raji cells and primary human B lymphocytes (PHB), we firstly demonstrate that HCV triggers phosphorylation of IκBα, up-regulates anti-apoptosis Bcl-2 family proteins, and enhances the protection of human B cells from Fas-mediated death. Moreover, E2-CD81 signaling increases CD81 and costimulatory molecules CD80 and CD86, and decreases complement receptor CD21. These results are helpful to understand the mechanisms involved in HCV-associated B cell lymphoproliferative disorders and weak neutralizing antibody production.

## Results

### Raji cells express HCV receptors CD81 and SR-BI

**Figure 1:**
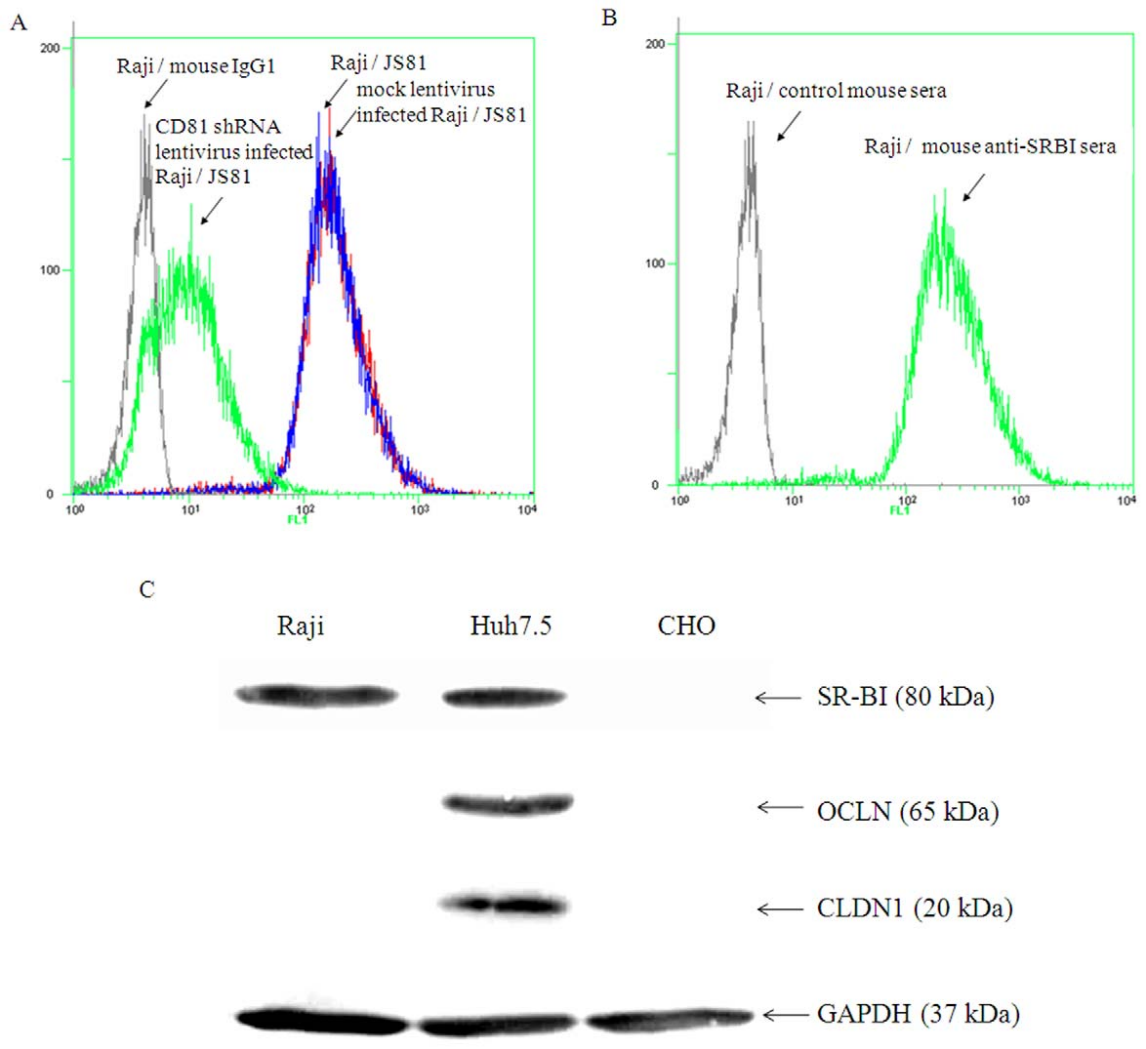
Expression of HCV receptors on Raji cells. (A). Expression of CD81 on naïve Raji cells, mock lentivirus infected Raji cells and CD81 shRNA lentivirus infected Raji cells were assayed by FACS. The primary antibodies used were anti-CD81 mAb JS81 and mouse isotype IgG1. (B) Expression of SR-BI on Raji cells. The primary antibodies used were mouse anti-SR-BI sera and control mouse sera. (C). Lysates of Raji, Huh7.5 and CHO cells were analyzed for expression of SR-BI, CLDN1 and OCLN by immuno-blotting. The primary antibodies used were mouse anti-human SR-BI, rabbit anti-human CLDN1 and mouse anti-human OCLN.

CD81, SR-BI, claudin-1(CLDN1) and occludin (OCLN) are considered to be the necessary surface factors for HCV infection [23]. CD81 could be detected on surface of naïve Raji cells, and was down-regulated by 95% after the cells infected with a lentivirus containing CD81-shRNA (Fig. 1A). Expression of SR-BI was detectable by FACS and Western blotting analysis (Fig. 1A, 1B). CLDN1 and OCLN could not be detected by Western blotting (Fig. 1B). Quantitative real-time RT-PCR was performed to determine mRNAs of CLDN1 and OCLN in Raji cells, and both were undetectable (data not shown). Huh7.5 cells express all of the receptors and CHO cells do not express any one of these molecules (CD81 expression on Huh7.5 and CHO cells assayed by FACS were not shown).

### CD81 mediates HCV E2 protein binding to Raji cells

**Figure 2:**
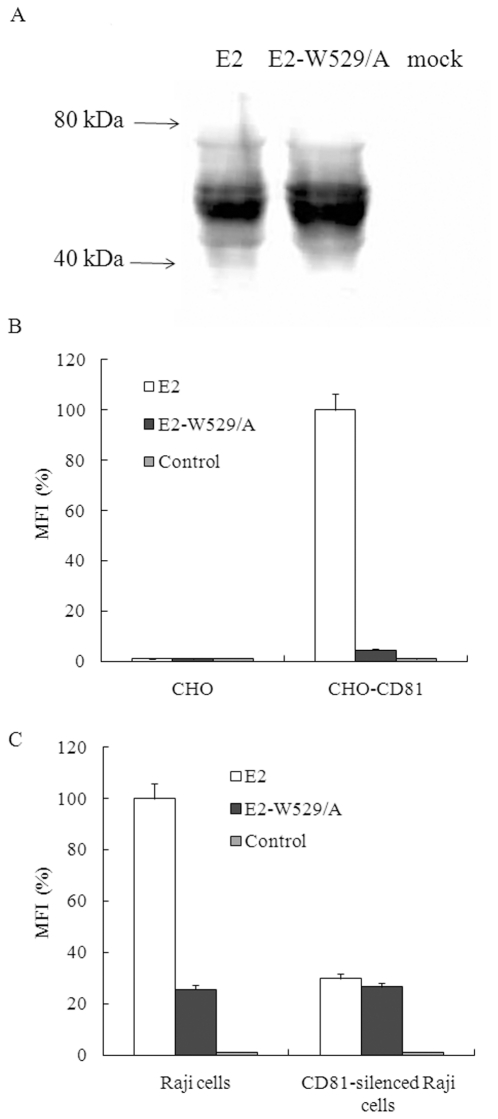
The role of CD81 in mediating HCV E2 binding to Raji cells. (A). 293T cells were transfected with HCV E2 expression plasmid, E2-W529/A expression plasmid, or mock plasmid, respectively. The cells were lysed at 72 h post-transfection and expression of E2 protein was analyzed using immuno-blotting. (B). The binding of cell extract containing HCV E2 protein with naïve or CD81 expression plasmid transfected CHO cells was measured using a FACS-based assay. E2 binding was expressed as the percentages of mean fluorescence intensity (MFI) relative to that of wild type E2 to CHO-CD81. Results are the means + standard deviations of three independent experiments. (C). The binding of cell extract containing HCV E2 protein with naïve or CD81-silenced Raji cells was measured using a FACS-based assay. E2 binding was expressed as the percentages of mean fluorescence intensity (MFI) relative to that of wild type E2 to Raji cells. Results are the means + standard deviations of three independent experiments.

It has been reported that the amino acid residue W529 in HCV E2 protein is essential for CD81 binding [24]. A mutant protein E2-W529/A was prepared, in which the tryptophan residue was replaced by an alanine (Fig. 2A). CD81 was detectable on CHO cells after transfected with CD81 expression plasmid (data not shown). Based on the analysis using this cell model, the CD81 binding activity of the mutant E2 protein is less than 5% of that the wild type protein (Fig. 2B). The E2 protein also binds to Raji cells, while the binding activity of mutant E2 decreased to about 26% of that the wild type protein (Fig. 2C). Compared with the binding activity of wild type E2 to naïve Raji cells, that of wild type E2 and the mutant E2 to CD81-silenced Raji cells decreased to 29% and 26%, respectively (Fig. 2C). These results indicate that CD81 plays a major role in mediating E2 binding to Raji cells, and other molecules, such as SR-BI, may also participate in the interaction between E2 and Raji cells.

### HCVpp and HCVcc fail to infect Raji cells

**Figure 3:**
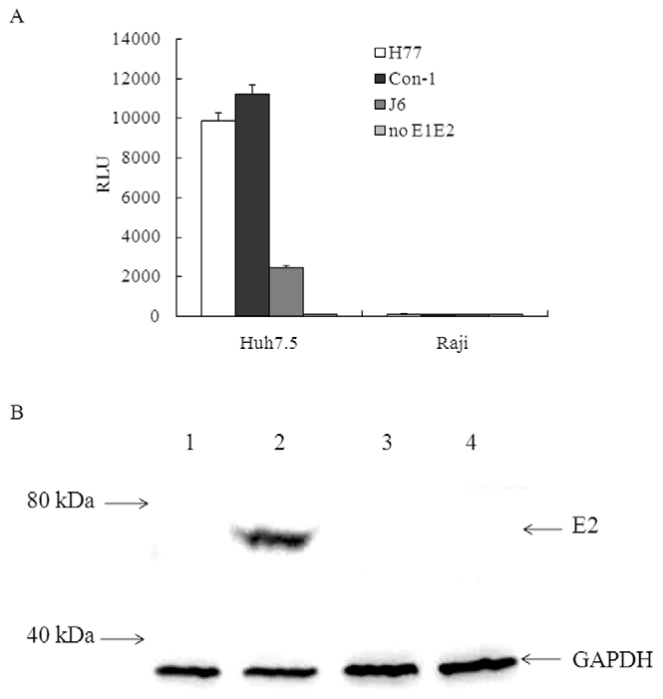
HCVpp and HCVcc infection of Huh7.5 and Raji cells. (A). HCV pp of 1a (H77 strain), 1b (Con-1 strain) and 2a (J6 strain) genotypes were used to infect Huh7.5 cells and Raji cells. At 72 h postinfection, the cells were lysed and then luciferase activity was determined using the Bright Glow Luciferase Assay System (Promega) and expressed as relative light units (RLU). Values are the means + standard deviations of three independent experiments. (B). Lysates of Huh7.5 cells and Raji cells infected with HCVcc were detected for E2 protein expression by immune-blotting. 1, Huh7.5 cells; 2, HCVcc infected Huh7.5 cells; 3, Raji cells; 4, HCVcc infected Raji cells.

The major site of HCV replication is the liver in host. However, it was reported that HCV RNA was detectable in peripheral blood mononuclear cells of infected individuals [25], [26], [27], [28], [29], [30]. Recently, some studies demonstrated that primary B cells or B cell lines are not permissive to HCV based on HCVpp and HCVcc models [31], [32]. In our observation, all of the tested pseudo particles, including that of H77 strain (1a subtype), Con-1 strain (1b subtype) and J6 strain (2a subtype), can infect Huh7.5 cells, but not infect Raji cells (Fig. 3A). The expression of E2 protein could be detected in Huh7.5 cells infected with the J6/JFH1 chimeric HCVcc, but could not be detected in Raji cells even incubated Raji cells with a higher dosage of virus (Fig. 3B). The negative-strand RNA of HCV was also determined by RT-PCR, it could be detected in the total RNA prepared from HCVcc infected Huh7.5 cells, but could not be detected in that from HCVcc infected Raji cells (data not shown).

### E2-CD81 engagement triggers phosphorylation of IκBα and up-regulates expression of NF-κB

**Figure 4:**
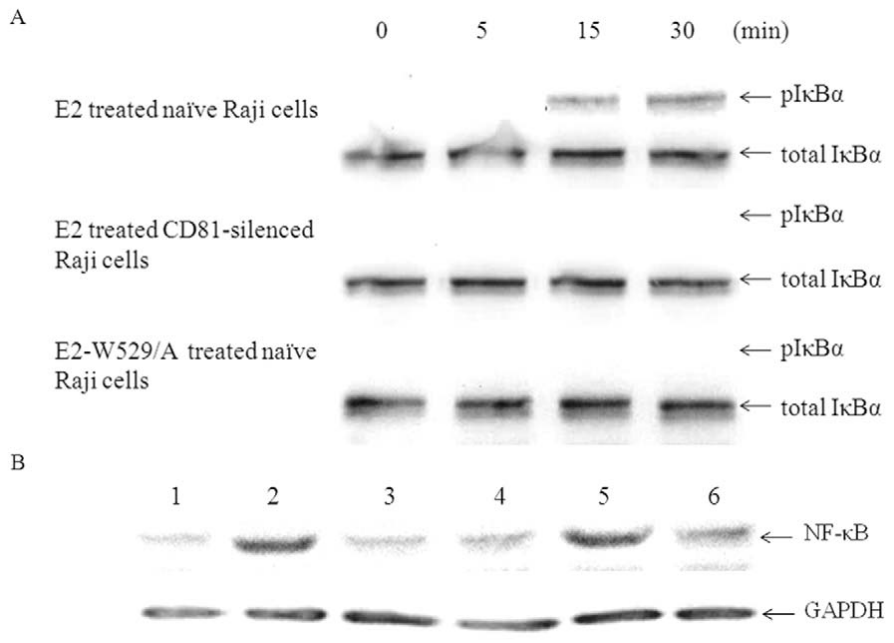
E2-CD81 engagement activates phosphorylation of IκBα and increases expression of NF-κB. (A). Proteasome inhibitor MG-132 treated naïve or CD81-silenced Raji cells were added to wild type E2 or E2-W529/A coated plates, at the indicated time points (minutes), the cells were lysed and the lysates were subject to Western blot analysis with anti-phospho-IκBα mAb and anti-total IκBα mAb. (B). Raji cells were cultured in HCV E2 protein coated plates or incubated with HCVcc, three days later, the cells were lysed, and then NF-κB in the lysates were analyzed using immuno-blotting, the ratios were obtained of the densitometric intensity of NF-κB band relative to the loading control GAPDH band. 1, Naïve Raji cells; 2, E2 treated Raji cells; 3, E2 treated CD81-silenced Raji cells; 4, E2-W529/A treated Raji cells; 5, HCVcc treated Raji cells; 6, HCVcc treated CD81-silenced Raji cells.

NF-κB transcription factor is a key regulator of B cell survival during the differentiation and activation of B cells by antigens or mitogens [33]. We dissected the possible CD81-mediated activation of NF-κB by E2 or HCV treatment. Raji cells were pretreated with proteasome inhibitor MG-132 (Merck), for this reagent can block the degradation of phosphorylated IκBα and consequently makes this factor easier to be detected [34]. As shown in Fig. 4A, phosphorylated IκBα could be detected at 15 min after E2 stimulation. Expression of NF-κB increased after treatment with E2 or HCVcc (Fig. 4B). Neither E2 treated CD81-silenced Raji cells, nor mutant E2-W529/A treated Raji cells produced these reactions, and the expression of NF-κB in CD81 silenced Raji cells treated with HCVcc was not increased (Fig. 4B).

### E2-CD81 engagement protects Raji and PHB cells from Fas-mediated death

**Figure 5:**
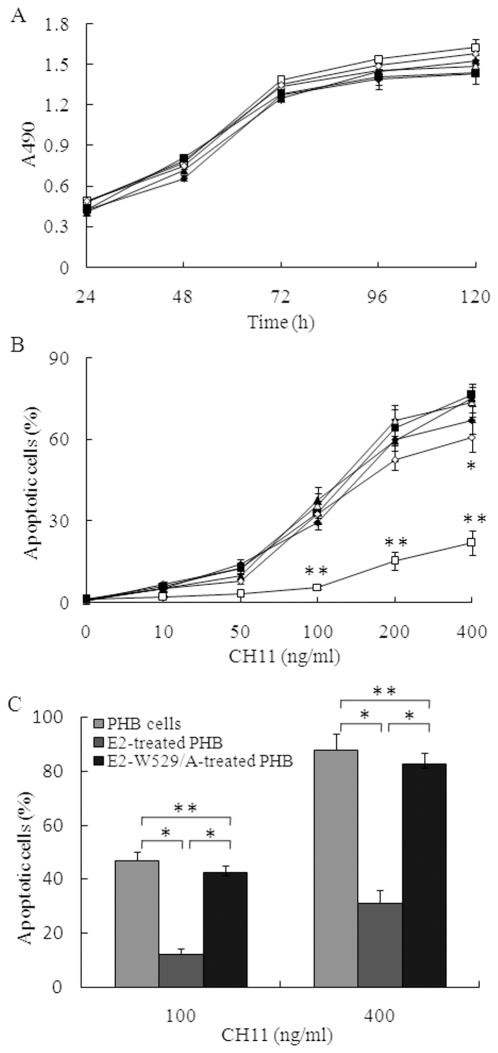
E2 blocks Raji cells apoptosis induced by anti-Fas antibody. (A). Raji cells or CD81-silenced Raji cells were placed in 96-well plates coated with or without HCV E2 protein, cell viability was measured by MTS assay at various time courses. Data represent the means ± standard deviations of triplicate determinations. The treatments of the cells were: Raji cells cultured in 96 wells without coating with HCV E2 protein (open triangles), CD81 silenced Raji cells cultured in 96 wells without coating with HCV E2 protein (filled triangles), E2-treated Raji cells (open squares), E2-W529/A-treated Raji cells (filled squares), E2-treated CD81 silenced Raji cells (open diamonds), E2-W529/A-treated CD81 silenced Raji cells (filled diamonds). (B). Raji cells or CD81-silenced Raji cells were cultured in 96-well plates coated with or without HCV E2 protein for 24 h, and then incubated with CH11 at various concentrations for 5 h. Apoptotic cells were measured by Hoechst 33342 staining. Data points represent the means ± standard deviations of triplicate determinations. The treatments of the cells were described above. Student's *t* test was used to determine the statistical significance. Double asterisks, *p*<0.001 relative to other cell-treatment combinations. Asterisk, *p*<0.05 relative to the CD81 silenced Raji cells without treatment with E2 protein. (C). PHB cells were cultured in HCV E2 protein pre-coated 96-well plates for 24 h, and then incubated with CH11 at concentrations of 100 or 400 ng/ml. Apoptotic cells were measured after 5 h. Double asterisks, *p*>0.05. Asterisk, *p*<0.001.

To observe whether E2 binding is able to enhance Raji cells' proliferation, we detected the proliferation of Raji cells after E2 stimulation. Under present conditions, E2 protein did not show obvious effect on the proliferation of Raji cells (Fig. 5A). ForPHB cells, similar results were observed (data not shown). Given the fact that E2-CD81 engagement can activate phosphorylation of IκBα and up-regulat expression of NF-κB, we next examined whether E2 can protect B cells from Fas-mediated death. We observed that treatment of Raji cells or PHB cells with anti-Fas CH11 resulted in significant apoptosis in a dose-dependent manner, and the wild type E2 protein, not the mutant E2-W529/A, strongly augmented the protection against apoptosis and enhanced cell viability (Fig. 5B and 5C). Under stimulation with the mAb at a concentration of 400 ng/ml, E2 protein also inhibited anti-Fas induced cell death in CD81-silenced Raji cells significantly (Fig. 5B). This may be partly owing to the residual CD81 expression on the cell surface.

### E2-CD81 engagement regulates protein expression of Bcl-2 family

**Figure 6:**
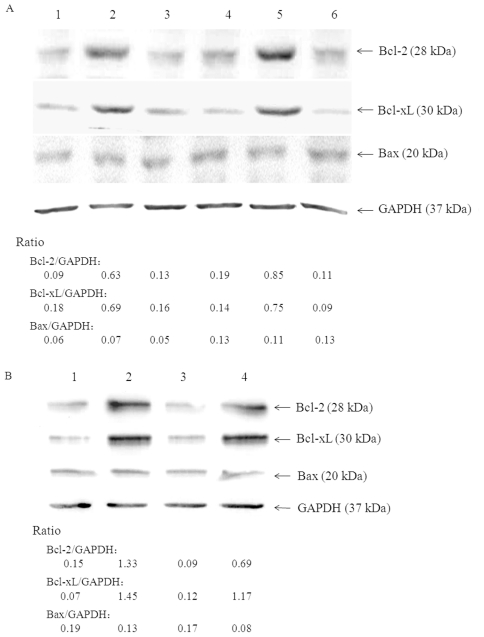
Effect of E2-CD81 engagement on expression of Bcl-2 family proteins. Raji cells (A) and PHB cells (B) were treated with HCV E2 protein or HCVcc as described above, three days later, cell lysates were prepared and Bcl-2, Bcl-xL and Bax were determined by Western blot analysis, the ratios were obtained of the densitometric intensity of anti-apoptotic or pro-apoptotic protein band relative to the loading control GAPDH. A: 1, Naïve Raji cells; 2, E2 treated Raji cells; 3, E2 treated CD81-silenced Raji cells; 4, E2-W529/A treated Raji cells; 5, HCVcc treated Raji cells; 6, HCVcc treated CD81-silenced Raji cells. B: 1, untreated PHB cells; 2, E2 treated PHB cells; 3, E2-W529/A treated PHB cells; 5, HCVcc treated PHB cells.

The proteins of Bcl-2 family play essential roles in the control of activation induced B cells apoptosis, and the transcription factor NF-κB regulates the expression of several anti-apoptotic gene products of this family [35]. It is possible that E2 induced B cells resistant phenotype to anti-Fas may correlate with the expression of these proteins. When assayed at 72 h after treatment, as showed in Fig. 6A, E2 elevated the expression of the anti-apoptotic proteins Bcl-2 and Bcl- xL in Raji cells, and did not affect the expression of Bax. This effect is exclusive to E2-CD81 binding, for the expression of these proteins were unchanged in both mutant E2-W529/A treated Raji cells and E2 treated CD81-silenced Raji cells. Expression of Bcl-2 and Bcl-xL was also up-regulated in HCVcc treated Raji cells, but not in HCVcc treated CD81 silenced Raji cells (Fig. 6A). A similar profile but higher expression levels of both Bcl-2 and Bcl-xL in Raji cells were observed at 24 h after treatment with E2 or HCVcc (data not shown). For PHB cells, the expression of Bcl-2 and Bcl- xL were also up-regulated by treatment with E2 protein and HCVcc, but not by the mutant E2 protein (Fig. 6B).

### E2-CD81 engagement modulates B lymphocyte activation markers

**Figure 7:**
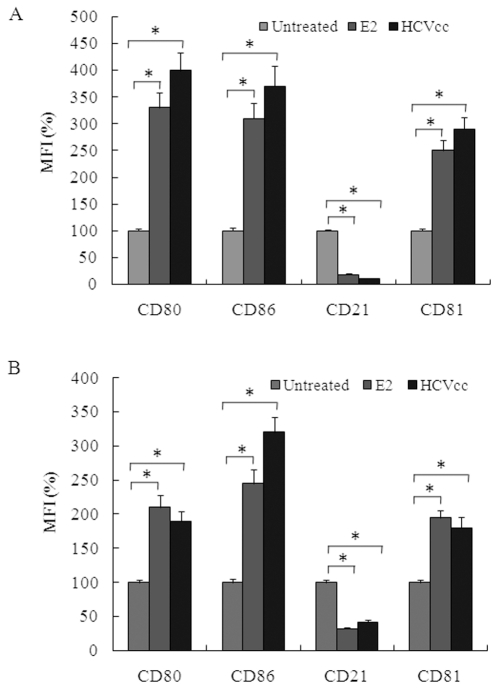
Effect of E2-CD81 engagement on expression of CD80, CD86, CD21 and CD81 on Raji and PHB cells. Raji cells (A) and PHB cells (B) were treated with HCV E2 protein or HCVcc, and the expressions of CD80, CD86, CD21 and CD81 were measured using a FACS-based assay. The mean fluorescence intensity (MFI) relative to untreated cells was calculated. Results are the means + standard deviations of three independent experiments. Asterisk, *p*<0.001.

Chronic HCV infection is often associated with the activation of B lymphocytes, and some studies indicated that E2-CD81 interaction may be responsible for this activation [14]. We found that the expressions of costimulatory molecules CD80 and CD86 on Raji cells and PHB cells were up-regulated, the expression of complement C3 receptor CD21 was down-regulated, and the expression of CD81 itself was elevated after treatment with E2 or HCVcc (Fig. 7).

## Discussion

In the present study, we demonstrated that HCV can modulate the activation, survival and immunological phenotype of Raji cells via E2-CD81 engagement, which may be related with B lymphocyte disorders and weak neutralizing antibody response in HCV patients.

It has been proposed that HCV infects B cells, which may lead to clonal B cell expansions. CD81, SR-BI, CLDN1 and OCLN have been proved to be necessary for HCV infection [23]. However, B cells in peripheral blood lack necessary HCV entry receptors and do not support HCV replication [32]. Our findings showed that the expressions of CD81 and SR-BI were detectable on Raji cells, but CLDN1 and OCLN were undetectable. Three strains of HCVpp with high infectivity to Huh7.5 cells failed to infect Raji cells, and there was no evidence showing HCVcc could infect Raji cells. These data suggest that HCV viral particles rarely infect B cells, at least under experimental conditions *in vitro,* although they may be able to bind with B cells via envelope proteins-cellular receptors interaction.

For the costimulatory role of CD81 on B cells, E2-CD81 binding is suggested as a contributory factor in the pathophysiological process leading HCV infection to B-cell clonal expansion [14]. But we did not observe obvious enhancement of E2 protein on proliferation of Raji cells and PHB cells under the present conditions. We think it is possible that the amount of E2 immobilized onto the culture plates is not sufficient to enhance the cell proliferation or more time is required to observe the effect of E2 protein on cell proliferation. Complement-binding of CD21/CD19/CD81 acts a role in enhancing protection of human B cells from Fas-mediated apoptosis [36], [37]. We found that treatment of Raji cells or PHB cells with CH11 anti-Fas mAb led to significant cell death, and E2 protein efficiently diminished cell death. The mutant E2-W529/A, which fails to bind with CD81, did not protect cells from death. Treatment of CD81-silenced Raji cells with E2 protein also showed no protective effect.

B cells are susceptible to mitochondria- and receptor-initiated death at various stages of peripheral differentiation and during immune responses, which plays an important role in maintaining homeostatic control of B lymphocytes [38], [39]. The transcription factor NF-κB enhances cell viability by activating genes that counteract both mitochondria- and receptor-initiated death pathways [33]. Bcl-2 family proteins that consist of anti-apoptotic and pro-apoptotic members are important regulators of apoptosis, which may be either death antagonists (e.g. Bcl-2 and Bcl-xL) or death agonists (e.g. Bax, Bad and Bak), the balance between these two types of Bcl-2 family members has been reported to partly control cell fate [40]. In the present study, E2-CD81 engagement triggered phosphorylation of IκBα and increased expression of NF-κB and NF-κB target genes Bcl-2 and Bcl-xL. A higher over-expression rate of Bcl-2 was reported in HCV patients with cryoglobulinemia (MC) compared those without MC, with a further increase in patients with non-Hodgkin lymphoma (NHL) [41], [42]. Moreover, antiviral treatment led to a decrease in Bcl-2 expression, which may further support the relationship between HCV infection and induction of Bcl-2 over expression [43]. A recent report indicated that mature activated B cells in patients with chronic HCV infection are intrinsically resistant to apoptosis, and expression of Bcl-2 in these cells were commonly elevated [44], [45]. Our results indicated that E2-CD81 engagement activates transcription factor NF-κB, which then increases the expression of Bcl-2 proteins and in turn enhances the survival of B cells and protects B cells from apoptosis. This possibility is supported by the observation that improvement of mixed cryoglobulinemia and non-Hodgkin lymphoma in chronic HCV patients after interferon therapy, however, no HCV proteins or HCV genome was able to be detected in villous splenic lymphoma cells in these patients prior to treatment [13].

It is reported that peripheral B cells from the majority of hepatitis C patients expressed elevated levels of B lymphocyte activation markers and a great number of non-specific activation of T cells infiltrated in liver, and the latter is considered an important cause of hepatocyte damage [14], [46]. In the present study, both E2 protein and HCVcc conferred Raji cells and PHB cells more activated phenotype by increasing the expressions of CD80, CD86, which are consistent with the observation that E2 promoted Raji cells to secret TNF-α [16]. Since activated B cells gain enhanced ability to stimulate T cells, we think E2 binding to CD81 on B cells should be involved in non-specific activation of T cells.

CD21-mediated complement recognition acts as an important role for B cells' response to specific antigens [9]. We found that E2 protein and HCVcc significantly decreased CD21 expression on Raji cells and PHB cells. This phenomenon is also consistent with the activation and maturation phenotype of B cells, which display decreased expression of CD21 [47]. If E2 in deed lowers CD21 expression *in vivo*, which would make B cells lose the ability of capturing opsonized antigen-complement C3d complex, and consequently decrease B cells' response to antigen-BCR engagement. Thus, it is possible that E2-CD81 engagement inhibits antibody response to E2 protein. It is interesting that CD81 itself was elevated by E2 or HCVcc treatment, which may act as a positive feedback between E2 engagement and B cells activation, so as to facilitate the establishment of HCV chronic infection and the progress of B-cell disorders. In fact, the expression of CD81 is increased on circulating B cells from HCV infected individuals, and decreased significantly in patients responded to IFN therapy [48], [49].

Although few existing characterized viral clones that can replicate in vitro have consistently failed to infect human B cells, some groups have detected HCV RNA in other lymphoid cells, including B- and T-lymphocytes, monocytes, and dendritic cells [25], [26], [27], [28], [29], [30]. A B-cell line (SB) established from an HCV-infected non-Hodgkin's B-cell lymphoma was reported to produces HCV particles that can further infect B- and T-lymphocytes in vitro [50], [51]. These data hint lymphotropism of HCV in natural infection may be possible. The data here strongly suggest that HCV may interfere with B cells independent on HCV replication in cells.

Together, the present study indicates that E2-CD81 engagement plays a role in activating B cells, protecting B cells from activation-induced cell death, and regulating immunological function of B cells. Therefore, the E2-CD81 engagement should be involved in the HCV associated B-cell disorders and insufficient neutralizing antibody response. These findings provide valuable insights into the development of therapeutic strategies against HCV infection and the related B-cell disturbance.

## Materials and Methods

### Cells

The B-cell line Raji, and the Chinese hamster ovary (CHO) cells (purchased from the Cell Bank of Shanghai Institutes for Biological Sciences, Chinese Academy of Sciences, Shanghai, China) were propagated in RPMI 1640 and DMEM media supplemented with 10% fetal bovine serum (Invitrogen), respectively. Huh7.5 cells, high permissive to HCV (provided by Dr.C.M. Rice, Rockefeller University, NY, USA), were propagated in DMEM supplemented with 10% heat-inactivated fetal bovine serum (Invitrogen) and 1% nonessential amino acids (Invitrogen).

### CD81 RNA interference

Human CD81 siRNA expression plasmid pGCsi-U6-CD81 siRNA5 was constructed in this laboratory [52], and used as a template for amplification of the siRNA expression cassette by PCR. The siRNA expression cassette was then inserted into lentivirus vector pLenti6 (Invitrogen). Lentivirus containing CD81 shRNA (short hairpin RNA) were generated using Power Lentiviral Support Kit (Invitrogen) according to the directions. Raji cells were infected overnight with the packaged lentivirus two times at an interval of three days, and then the expression of CD81 was assayed by a fluorescence-activated cell sorting (FACS) using a FACSCalibur instrument (Becton Dickinson).

### HCV E2plasmid constructs and protein expression

DNA sequence encoding carboxyl terminal truncated E2 protein (aa 364–661 in HCV polyprotein) of strain H77, genotype 1a (GenBank accession no. AF009606) was synthesized by overlap extension PCR using optimized codons of highly expressed mammalian genes, and the resulting DNA fragment was sequenced and inserted into pCI-neo plasmid (Promega). A mutant E2-W529/A, in which the 529th aa tryptophan was replaced by alanine, was prepared using Site-directed Gene Mutagenesis Kit (Stratagene), and then inserted into pCI-neo vector. The expression plasmids were transfected into 293T cells by using Lipofecatamine 2000 (Invitrogen), respectively. At 72 h after transfection, the cells were removed from the tissue culture dishes by phosphate-buffered saline (PBS)-EDTA treatment, resuspended in PBS supplemented with proteinase inhibitor cocktail (Roche), and lysed by ultrasonication. The lysates were centrifuged and the supernatants were removed and concentrated 10-fold by using Centricon ultrafiltration tube (Millipore).The expression of E2 and E2-W529/A was assessed with goat anti E2 polyclonal antibodies (Biodesign International) using Western blotting. HCV E2 protein in the lysates was normalized by using ELISA described previously [53], [54]. The E2 mAb H53 (provided by provided by Dr. J. Dubussion, Institut Pasteur, Lille, France) was used as a detective antibody. This antibody is not a neutralizing antibody, and does not interfere with the interaction between E2 and CD81 [53], [54].

### HCV E2 binding with CHO and Raji cells

The human CD81 expression plasmid was constructed in this laboratory, this plasmid and mock vector were separately transfected into CHO cells by using Lipofecatamine 2000 (Invitrogen). At 48 h after transfection, the expression of CD81 on CHO cells was assayed by FACS. The binding of HCV E2 protein with the transfectant CHO cells and Raji cells were measured using FACS-based assay [53], [55]. The cells were washed twice in PBS supplemented with 2% fetal calf serum and 0.05% NaN3 (washing buffer). Then, 5×10^5^ cells were incubated with crude cell extract containing E2 proteins or control cell extract for 1 h at room temperature in washing buffer and were washed twice with PBS. The cells were incubated for 1 h at 4°C with diluted polyclonal goat anti-E2. After incubation with FITC conjugated rabbit anti-goat IgG, E2 binding was quantified by flow cytometry (mean fluorescence intensity, MFI).

### HCV pseudoparticles (HCVpp) production and infection

HCVpp were generated as described previously [31]. Briefly, 293T cells were cotransfected with expression vector encoding the HCV envelope glycoproteins, gag/pol (pLP1), rev (pLP2), and transfer vector encoding the luciferase. HCV envelope expression plasmids encoding E1 and E2 glycoproteins of genotype 1a strain H77 (provided by Dr. F.L.Cosset, INSERM U758, Lyon, France), genotype 1b strain con-1 (provided by Dr. C. M.Rice, Rockefeller University, NY, USA), genotype 2a strain J6 (provided by Dr. C. M. Rice, Rockefeller University, NY, USA) were used. The culture supernatants containing HCVpp were harvested at 48 h after transfection, and filtered through 0.45-µm-pore-size membrane for infection use.

Target cells, Huh7.5, or Raji cells were seeded into 96-well plates at a density of 1×10^4^ cells/well and incubated overnight at 37°C. HCVpp supernatants were added 50 µl to each well, and incubated for 5 h. The supernatants were removed and the cells were incubated in regular medium for 72 h at 37°C. Cells were washed once with PBS and lysed with 50 µl of cell lysis buffer (Promega) per well. Luciferase activities were quantified using a Bright Glow Luciferase Assay System (Promega).

### Cell culture produced HCV (HCVcc) generation and infection

The plasmid pFLJ6/JFH1, containing the full-length chimeric HCV genomic cDNA of J6 and JFH-1 isolate and kindly provided by Dr. C. M.Rice (Rockefeller University, NY), was used to generate HCVcc as described previously [4]. Briefly, the RNA was transcribed from full-length genomes using the *in vitro* MEGAscript kit (Promega) and delivered into Huh-7.5 cells by electroporation. Viral stocks were obtained by harvesting cell culture supernatants at days 8–12 after transfection. The virus was concentrated by polyethylene glycol (PEG) precipitation and the viruspellet was resuspend in complete PRMI1640 medium. Infection was quantified by enumerating HCV E2 positive cells and was defined as the number of focus-forming units (FFUs).

Huh7.5 cells and Raji cells were seeded 5×10^5^ per well in 2 mL of media in 24-well plates, respectively. Meanwhile, HCVcc was added to Huh7.5 cells at a multiplicity of infection of 0.5 (2.5×10^5^ FFU), and to Raji cells at a multiplicity of infection of 2 (1×10^6^ FFU). The cell cultures were incubated at 37°C in an incubator at 5% CO_2_ atmosphere. Three days later, the cells were collected for FACS and Western blot assay.

### Assay of negative stand RNA of HCV using PCR

The total RNA was isolated from Huh7.5 and Raji cells using Rneasy mini kit (Qiagen) three days after HCVcc infection, and then negative HCV RNA strand was detected using RT-PCR. The procedure and specific primers are detailed as described previously [56].

### Isolation of human B lymphocytes

PBMCs from four healthy donors were isolated using standard ficoll density gradient centrifugation. Informed consent was obtained from the donors studied. The final pellet was resuspended in RPMI 1640 medium supplemented with 10% foetal calf serum. Enriched B cells were isolated by positive selection using magnetically labeled antibodies specific for human CD19 (Miltenyi Biotec, Bergisch Gladbach, Germany) according to the manufacturer's instructions. Briefly, 5 × 10^7^ PBMCs were incubated with 100 µl of CD19 Microbeads for 15 min at 4°C. These cells were passed through a positive-selection column. The purified B lymphocytes were stained with FITC-anti-CD19 mAb and then were sorted by flow cytometry with >97% purity.

### E2 coating and cell stimulation

HCV E2 protein was coated as described previously [7], [57], [58]. Briefly, the E2 mAb H53 was diluted to 10 µg/ml in carbonate buffer (15 mM Na_2_CO_3_, 35 mm NaHCO_3_, pH 9.6) and added to each well of 96 or 24-well plates. The plates were incubated overnight at 4°C, and then washed three times with phosphate-buffered saline (PBS) and saturated for 30 min at 37°C with complete RPMI1640 medium. The normalized cell extracts of 293T cells transfected with HCV E2 expression plasmids were added to the wells, and plates were incubated for 60 min at 37°C. After further washing with PBS buffer, Raji cells or PHB cells in complete medium were added to the coated plates, followed by incubation for various time periods as indicated in the experiments.

### Cell viability and apoptosis assay

Raji cells or PHB cells (2×10^3^ cells, 200 µL) were cultured in 96-well plates coated with or without HCV E2 protein, and incubated at 37°C in an incubator at 5% CO_2_ atmosphere. The MTS/PES solution (Promega) was used for cell viability assays according to the directions. 20 µL premixed solution of 3-(4,5-dimethylthazol-2-yl)-5-3-carboxymethoxy-phenyl)-2-(4-sulfophenyl)-2H-tetrazolium (MTS) and phenazine methosulfate (PMS) was added per well at hours 24, 48, 72, 96 and 120, and the cell cultures were continued for additional 4 h at 37°C for color development. Then the absorbance of the cell cultures at 490 nm was measured using a 96-well plate reader (Bioteck).

The anti-Fas mAb CH11 was used to induce cell apoptosis and the cell apoptosis was assayed using Hoechst 33342 staining as described previously [36], [59]. Briefly, cells (2×10^4^ cells, 200 µL) were incubated in E2 coated plates for 24 h, pulsed with anti-Fas mAb CH11 at diverse concentrations for 5 h, and then fixed with 4% paraformaldehyde/PBS, followed by staining with 300 µM Hoechst 33342 (Calbiochem). The number of normal and condensed nuclei was counted under fluorescent microscope. Apoptotic cells (%) were calculated as (condensed nuclei/total nuclei) ×100. The cell viability was assayed using MTS/PES and expressed as a percentage of absorbance in cells with indicated treatments to that with control treatment.

### Western blotting

Cell lysates were separated by sodium dodecylsulfate (SDS)-12.5% polyacrylamide gels electrophoresis (PAGE). Proteins were then electrophoretically transferred onto nitrocellulose membranes for immuno-blotting analysis. The primary antibodies used were: mouse anti-SR-BI (BD Pharmingen), rabbit anti-human CLDN1 (Cell Signaling), mouse anti-human OCLN (Invitrogen), goat anti-HCV E2 (Biodesign International), anti-phospho-IκBα mAb (Cell Signaling), anti-total IκBα mAb (Cell Signaling), rabbit anti-NFκB p65 (Cell Signaling), rabbit anti-Bcl-2 (Cell Signaling), rabbit anti-Bcl-xL (Cell Signaling), rabbit anti-Bax (Cell Signaling) and rabbit anti-GAPDH (Cell Signaling). The secondary antibodies were: peroxidase-conjugated anti-mouse IgG, anti-goat IgG and anti- rabbit IgG. Immunoreactivity was visualized with enhanced chemiluminescence (GE Healthcare).

To detect phosphorylated IκBα, Raji cells were pretreated with proteasome inhibitor MG-132 (Merck) at a concentration of 20 µM for 30 min at 37°C and then added to HCV E2 coated plates. After incubation of 0, 5, 15, or 30 min at 37°C, the cells were washed and lysed for Western blot analysis.

### Flow cytometry

Cells were washed with PBS containing 1% bovine serum albumin, resuspended in the same buffer, and then incubated with the following mAbs: anti-CD81 clone JS81 (BD Pharmingen), anti-CD80 (BD Pharmingen), anti-CD86 (BD Pharmingen), anti-CD21 (BD Pharmingen), and anti-SR-BI mouse sera (prepared by DNA immunization of Balb/C mouse with human SR-BI expression plasmid) for 30 min on ice, respectively. After washes, the cells were incubated with FITC-conjugated secondary antibodies for 30 min on ice, then washed and assessed using a FACSCalibur (Becton Dickinson).
